# Authors’ Response to Peer Reviews of “Machine Learning for Risk Group Identification and User Data Collection in a Herpes Simplex Virus Patient Registry: Algorithm Development and Validation Study”

**DOI:** 10.2196/28917

**Published:** 2021-06-11

**Authors:** Svitlana Surodina, Ching Lam, Svetislav Grbich, Madison Milne-Ives, Michelle van Velthoven, Edward Meinert

**Affiliations:** 1 Skein Ltd London United Kingdom; 2 Department of Informatics King’s College London London United Kingdom; 3 Institute of Biomedical Engineering Department of Engineering Science University of Oxford Oxford United Kingdom; 4 Centre for Health Technology University of Plymouth Plymouth United Kingdom; 5 Nuffield Department of Primary Health Sciences Medical Sciences Division University of Oxford Oxford United Kingdom; 6 Department of Primary Care and Public Health School of Public Health Imperial College London London United Kingdom

**Keywords:** data collection, herpes simplex virus, registries, machine learning, risk assessment, artificial intelligence, medical information system, user-centered design, predictor, risk

*This is the authors’ response to peer-review reports for "Machine Learning for Risk Group Identification and User Data Collection in a Herpes Simplex Virus Patient Registry: Algorithm Development and Validation Study."*

## Round 1

Thank you for your consideration of our manuscript [1] for publication in *JMIR Medical Informatics*. We have made amendments to the manuscript reflecting the valuable review comments [2,3] forwarded to us and feel the paper is now acceptable for publication. Please let us know if there is anything we can do further to improve our paper.

### Reviewer Z

#### General Comments

We thank the reviewer [2] for their time and consideration in the review of our manuscript.

1. Thank you for your feedback; the issues raised have been addressed as advised by reworking the *Introduction*, *Challenges*, and *Discussion* sections in order to improve the focus and clarity of the paper.

2. We agree that there was a lack of precision in specifying the challenges and overlap in the problems described. We have edited the list and the objectives to focus on data collection and user experience in the *Aims and Objectives* section.

The privacy and security considerations, while remaining a critical element of the registry development, are not directly addressed in our study and have been set aside for future work.

Control over one’s own data is linked to the amount of disclosed data (perceived and objective), so it is related to the objectives addressed by the algorithm.

3. Thank you for the comment. As this paragraph lacked clarity on the fact that the goal of using the ArthritisPower example was to illustrate the variety of approaches and the specificity of registry contexts, design approaches, and purpose, we have highlighted the differences and limitations of design studies for registries such as ArthritisPower.

4. In the *Introduction*, we have added the section *Review of Past Studies*, which narrows down the focus of our study and better places it in the context of past research.

5. In using the US National Health and Nutrition Examination Survey (NHANES) data set, we were guided by the NHANES data disclosure terms, which indicate that all data sets comply with the anonymization requirements and have been approved by the National Center for Health Statistics Research Ethics Review Board (previously the NHANES Institutional Review Board [IRB]; protocol #2018-01). Hence, since we only analyzed anonymized data without identification, no direct communication with the IRB was made. The guidance terms and conditions used can be found online [4].

We have added a corresponding comment to the *Database Used* subsection. The sentence was expanded.

#### Minor Comments

6. Pg 3, lines 3,4: The word “data” has been added.

7. Pg 3, line 7: We have updated the *Introduction* section with a few examples and a reference to our previous study, which focused on the unique challenges that a herpes simplex virus (HSV) registry design poses [5]. Paragraphs in the *Introduction* and *Discussion* sections have been expanded

8. Pg 6: Thank you for the comment; the criteria in the list have been edited to clearly differentiate between each point. Clarifications were also added. Bullet points were edited and expanded upon.

9. Pg 6, line 20: As JMIR uses the American Medical Association style guide, we edited the spelling to be consistent with American spelling. Spelling was changed in 17 instances.

10. Pg 7, lines 16,17: This ratio was chosen in order to keep the variance low and to leave enough data for training, and it is used as a standard split. A smaller training set was tested; however, it resulted in poorer performance. The text was clarified to add the rationale.

11. Pg 8: A sentence has been added to clarify the process and the scope of the paper.

12. Pg 9, lines 24-26: We have edited the paragraph to include the indication of the maximum possible number of questions (n=62) and predicted time to complete the full questionnaire. Moreover, we additionally reviewed the literature to estimate the expected improvement in drop-off rates and added this to the text. The maximum number of questions (n=62) has been added to the text, as well as an estimate of an average reduction in time that is needed to answer the questionnaire to generate a high-reliability risk group prediction.

13. Pg 10, line 1: The edit was made accordingly.

14. Pg 11, lines 13,14: We primarily relied on the result of our previous study [5], where semistructured reviews indicated these challenges and such links. In terms of quantitative evidence from the literature, it was hard to estimate the degree of possible improvements; the existing research highlights such complexity due to the multifactorial nature of “response burden,” but multiple research papers showed evidence of an improvement when using shorter questionnaires, with some cases of better retention among particular groups [6,7]. One future direction would be to test the actual change in real data once the model is trialed. Moreover, some of the sensitive questions (that are usually the best indications for HSV type 2) were removed at the data preprocessing stage, still obtaining a high accuracy for the model. The *Data Set Preparation* section has been expanded.

15. Pg 11, line 21: This has been changed to reflect the suggestion.

16. Pg 12, lines 5,6: Although the users are based in the United Kingdom and the study focused on the UK context, for the purposes of this study, we used a US data set due to the free access, sufficient size, and presence of extensive variables. Our research aims to lay the groundwork that would be applicable for patient data collection systems both among UK and US users. The *Intended User Journey* subsection has been edited.

17. Pg 12, line 22: A more detailed description has been added.

### Reviewer BK

#### General Comments

We thank the reviewer [3] for their time and consideration in the review of our manuscript. The issues raised have been addressed in view of the peer-review feedback provided.

#### Specific Comments

##### Major Comments

1. We have reviewed the guidelines and applied the following changes:

- Structure
- Changed *Background* to *Introduction*
- Heading format (removed numbering)
- Added titles for the multimedia appendices
- Shorten the paper by moving a figure into the *Multimedia Appendices* section
- Removed author-made abbreviations
- Edited title
- Edited the order of sections

2. In the *Introduction*, we have added the section *Review of Past Studies*, which narrows down the focus of our study and reviews studies applied to the classification problem in the context under consideration.

3. We have added the clarification that the split into the train and test subsets was done at random to ensure the data in both data sets were evenly distributed. We thank you for highlighting the missing details on preprocessing; this was separated into a subsection and clarified. Cross-validation: thank you, this indeed wasn't described in the text. We have now added a section describing these steps.

4. A matrix of confusion has been added.

5. Thank you for the suggestion; we have used GridSearchCV but did not mention it in the previous version of the paper. We have added a section describing these steps.

6. In the *Review of Past Studies* section, we have compared existing models with the proposed approach.

7. The code has been added to an open repository on GitHub and is now available online [8].

## Round 2

### Reviewer Z

#### Specific Comments

##### Major Comments

Thank you for your valuable review comments [2]. We have now worked on a copyediting review, addressing the UK English spelling instances among other issues.

##### Minor Comments

1-3. The text has been edited as suggested.

4. The labels have been added.

5-9. The text has been edited as suggested.
